# Strengthening government management capacity to scale up HIV prevention programs through the use of Technical Support Units: lessons from Karnataka state, India

**DOI:** 10.9745/GHSP-D-14-00141

**Published:** 2014-12-02

**Authors:** Sema K Sgaier, John Anthony, Parinita Bhattacharjee, James Baer, Vidyacharan Malve, Aparajita Bhalla, Vijaykumar S Hugar

**Affiliations:** a Bill & Melinda Gates Foundation, Global Development Group, and University of Washington, Department of Global Health, Seattle, WA, USA.; b India Health Action Trust, Karnataka Technical Support Unit, Bangalore, India.; c India Health Action Trust, Bangalore, India.; d Independent Consultant for the Bill & Melinda Gates Foundation, New Delhi, India.; e Karnataka State AIDS Prevention Society, Bangalore, India.

## Abstract

Scaling up HIV prevention programming among key populations (female sex workers and men who have sex with men) has been a central strategy of the Government of India. However, state governments have lacked the technical and managerial capacity to oversee and scale up interventions or to absorb donor-funded programs. In response, the national government contracted Technical Support Units (TSUs), teams with expertise from the private and nongovernmental sectors, to collaborate with and assist state governments. In 2008, a TSU was established in Karnataka, one of 6 Indian states with the highest HIV prevalence in the country and where monitoring showed that its prevention programs were reaching only 5% of key populations. The TSU provided support to the state in 5 key areas: assisting in strategic planning, rolling out a comprehensive monitoring and evaluation system, providing supportive supervision to intervention units, facilitating training, and assisting with information, education, and communication activities. This collaborative management model helped to increase capacity of the state, enabling it to take over funding and oversight of HIV prevention programs previously funded through donors. With the combined efforts of the TSU and the state government, the number of intervention units statewide increased from 40 to 126 between 2009 and 2013. Monthly contacts with female sex workers increased from 5% in 2008 to 88% in 2012, and with men who have sex with men, from 36% in 2009 to 81% in 2012. There were also increases in the proportion of both populations who visited HIV testing and counseling centers (from 3% to 47% among female sex workers and from 6% to 33% among men who have sex with men) and sexually transmitted infection clinics (from 4% to 75% among female sex workers and from 7% to 67% among men who have sex with men). Changes in sexual behaviors among key populations were also documented. For example, between 2008 and 2010, the proportion of surveyed female sex workers in 9 districts reporting that they used a condom at last intercourse rose from 60% to 68%; in 6 districts, the proportion of surveyed men who have sex with men reporting that they used a condom at last anal sex increased from 89% to 97%. The Karnataka experience suggests that TSUs can help governments enhance managerial and technical resources and leverage funds more effectively. With careful management of the working and reporting relationships between the TSU and the state government, this additional capacity can pave the way for the government to improve and scale up programs and to absorb previously donor-funded programs.

## Abstract (precis)

A Technical Support Unit of managerial and technical experts, embedded in but distinct from the government, provided support in 5 key areas: strategic planning; monitoring and evaluation; supportive supervision; training; and information, education, and communication. This model likely contributed to effective and rapid scale up of Karnataka state's HIV prevention program. A clear mandate, close collaboration, and well-defined roles were keys to success.

## INTRODUCTION

Scaling up national public health programs requires governments to have strong coordination, management, and technical capacity. A wide range of skills is needed to develop and issue policies, strategies, and implementation guidelines; manage and administer funds; procure commodities; manage systems and human resources; implement programs; provide technical and managerial support to programs; and monitor and evaluate them. However, in most settings, resource-limited or not, governments are often unable to act effectively across such a wide spectrum of program needs, because they lack either the human resources and the broad range of needed skills or the appropriate structures and processes. The Government of India's HIV prevention programming illustrates both this issue and a potential solution: insourcing expertise and skills from the private and nonprofit sector through a Technical Support Unit (TSU) to provide rapid, flexible support in a way that government systems cannot always manage.

In this article, we describe the background to the formation of TSUs in India, and show how a TSU was formed in the state of Karnataka, how it worked, and what the results were. This article consolidates the experience of stakeholders involved in the design and implementation of technical assistance in India, including government, donors, and implementing partners.

## SCALING UP HIV PREVENTION IN INDIA

The Government of India conceptualizes its HIV/AIDS response in 5-year strategic plans called the National AIDS Control Programme (NACP).^1^ The government's third such program (NACP-III), which ran from 2007 to 2012, prioritized HIV prevention among key populations, including female sex workers and men who have sex with men, as one of its main pillars, because of the disproportionately high rate of infection within these groups and the possibility of HIV transmission from members of these groups to members of the more general population.^2^ Two-thirds of the budget was allocated to prevention programming, with the goal of scaling up the number of prevention interventions to ensure high levels of coverage.^3^^,^^4^ In the context of NACP-III, scaling up referred to increasing coverage of key populations so that they all had access to HIV prevention programs as well as to increasing the quality of these programs.

**Figure 1.**
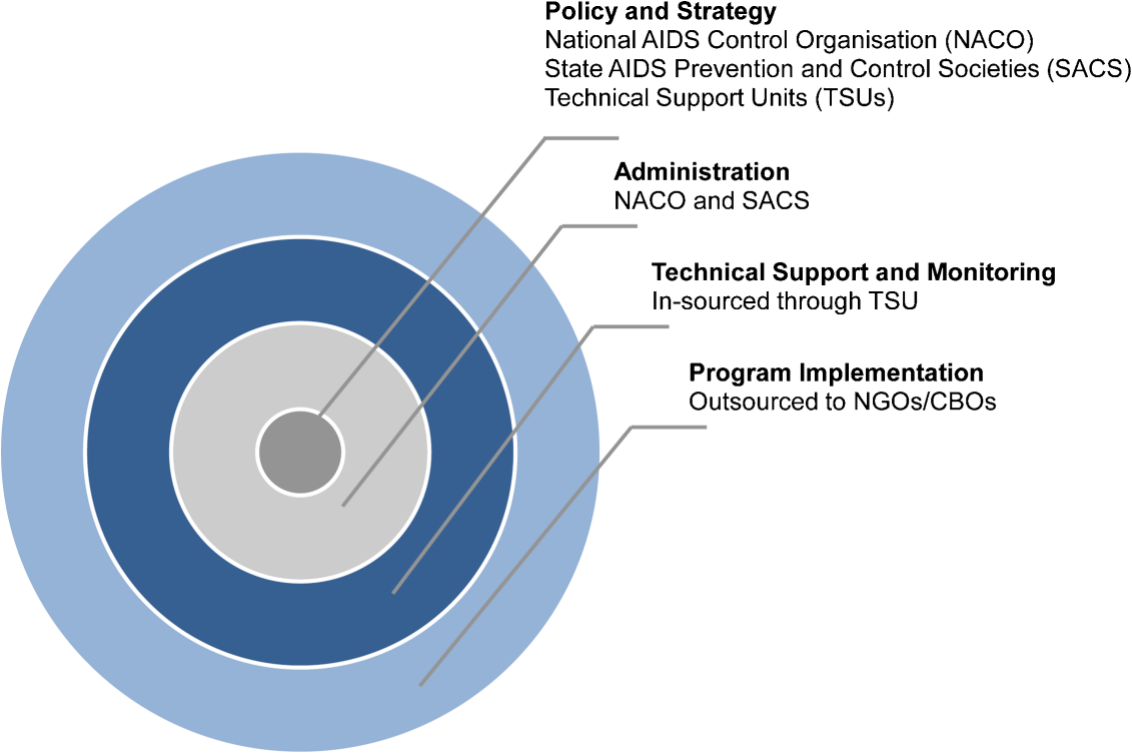
India's Strategy for Sourcing Capabilities to Accelerate Scale Up of the HIV Prevention Program Among Key Populations Abbreviations: CBOs, community-based organizations; NGOs, nongovernmental organizations.

To facilitate this scale up, NACP-III called for decentralized planning and implementation of the program. While the national body (the National AIDS Control Organisation, or NACO) was responsible for setting policies and guidance, responsibility for implementation in each of the then 28 Indian states fell to each State AIDS Prevention and Control Society (SACS). As part of the national strategy, program implementation was outsourced to nongovernmental organizations (NGOs) and community-based organizations (Figure 1), which were directly contracted by the SACS. These were known as intervention units. Outsourcing implementation to NGOs in the health sector is common in India and not limited to HIV/AIDS programs. The SACS, however, was ultimately responsible for the state's performance and for ensuring that the response achieved adequate scale and coverage.

A key strategy of the national government under NACP-III was to strengthen program management and technical capacity of the SACS.^2^^,^^5^ A lesson learned during the previous national strategy period, NACP-II (2000–2006), was the importance of supportive supervision—direct and intensive technical and management support of interventions—and constant capacity building of the SACS and NGOs to improve the coverage and quality of services. The state government was well-equipped to perform its core skills of administration, procurement, release of funds, and audits. However, it lacked key technical and program management skills to implement the programs. The SACS, in particular, lacked both the human resources and the skills needed to manage such programs, particularly as delivered by a network of intervention units.

In the early 2000s, some Indian states had experimented with Project Management Units—externally funded groups of experts who helped government teams with program design, evaluation, and reporting. NACO recognized the value of these units, but they had been formed on an ad hoc basis, so the government decided to standardize the approach. NACP-III conceptualized Technical Support Units (TSUs), including roles and responsibility, structure, and skills, and it mandated their formation for most states across the country to help with scaling up HIV prevention programming (Figure 1). Most TSUs were funded directly by the federal government, while a few were supported by donor agencies.

The national government mandated state AIDS agencies to form Technical Support Units to strengthen their management and technical capacity.

## THE MANDATE, FUNCTION, AND STRUCTURE OF TSUs

**Figure 2.**
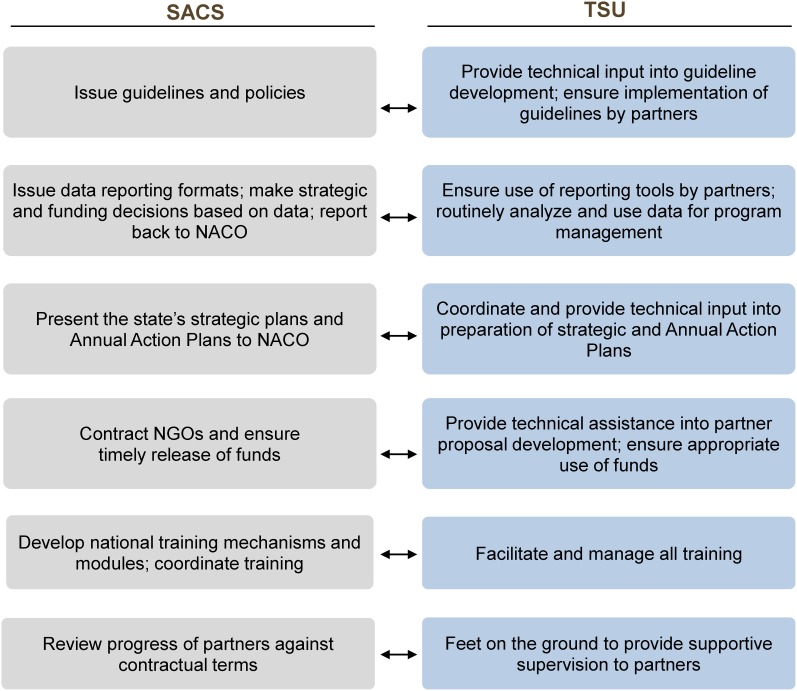
Complementary Roles of the State AIDS Prevention and Control Society and Technical Support Unit Abbreviations: NACO, National AIDS Control Organisation; NGOs, nongovernmental organizations; SACS, State AIDS Prevention and Control Society; TSU, Technical Support Unit.

TSUs are management units contracted by NACO and embedded within the SACS to provide technical and management support to the implementation of HIV prevention programs among key populations. Each TSU is comprised of experts from the private and nonprofit sector. The TSU is responsible for all technical aspects of the program, while administrative functions (procurement and finances) remain with government staff. The TSU ensures that national policies and strategies are implemented at the state level by working in close coordination with the SACS and providing capacity building and direct implementation support to intervention units. While the overall aim of the TSU is the same as that of the SACS—to support HIV prevention among key populations—it focuses on specific areas to ensure program scale and coverage and complements the skill set found within the SACS (Figure 2). It also ensures that program planning and implementation are evidence-driven and improves program accountability by strengthening monitoring and evaluation (M&E) systems.

Each Technical Support Unit is comprised of experts from the private and nonprofit sector with a complementary skill set to that found in the state government.

**Figure 3.**
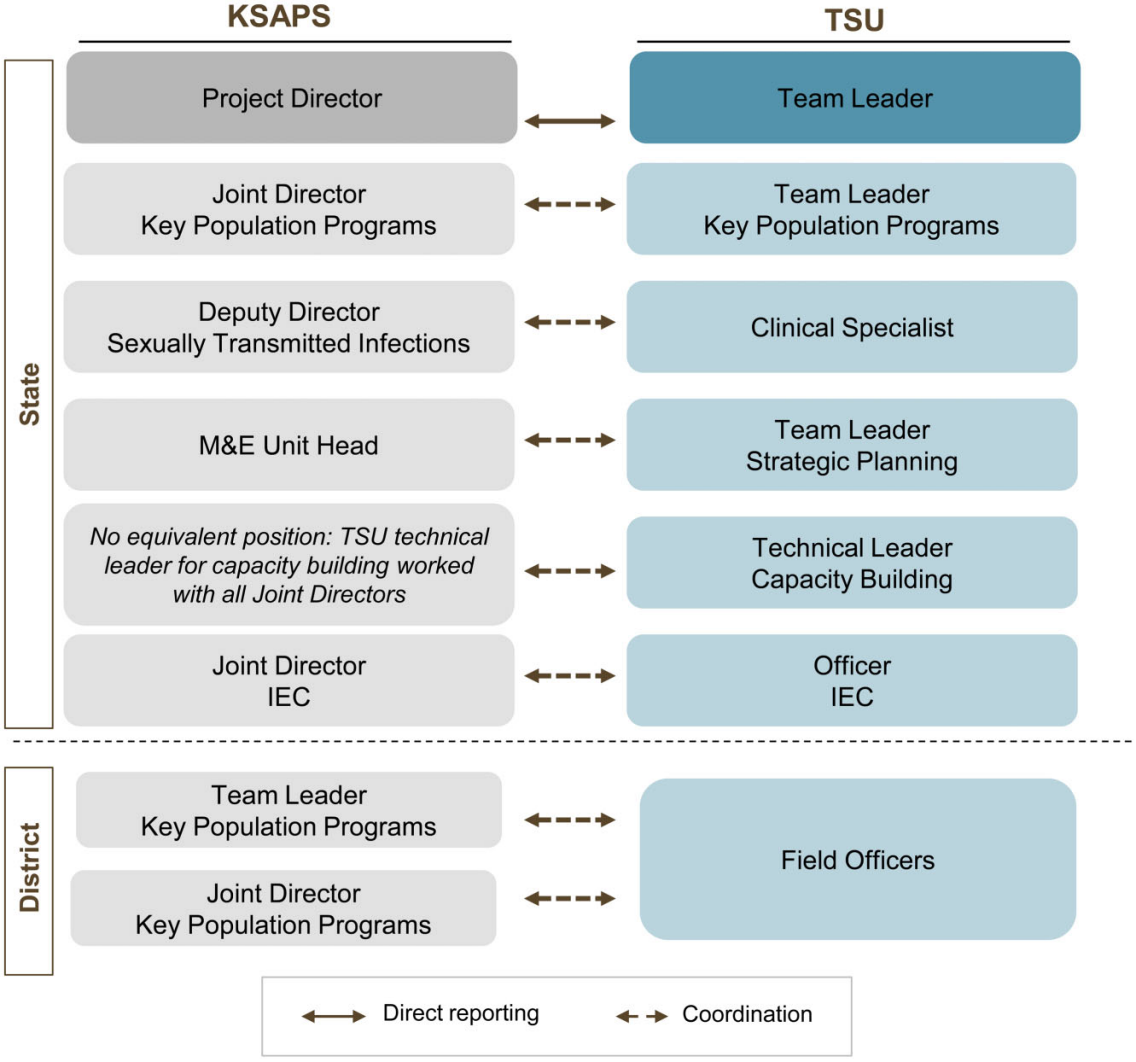
Coordination Between the Karnataka State AIDS Prevention Society and the Technical Support Unit Abbreviations: IEC, information, education, and communication; KSAPS, Karnataka State AIDS Prevention Society; M&E, monitoring and evaluation; TSU, Technical Support Unit.

The TSU model is different from a traditional model of providing technical support to governments by seconding staff. Crucially, the staff of each TSU are not part of the SACS itself; instead, each TSU is separate from—although closely interconnected with—the preexisting government department (Figure 3). The TSU has its own team leader, who is responsible for day-to-day operations but accountable to the head of the government department. This separate structure enables the unit to respond quickly to changing conditions on the ground.

## THE LOCAL CONTEXT: KARNATAKA STATE

Karnataka, with a population of 61 million, is one of India's 6 high-priority states for HIV prevention. In 2007, HIV prevalence among attendees of public antenatal clinics in Karnataka was 0.86% (a proxy for overall HIV prevalence).^6^ The epidemic is largely driven by unprotected sex between approximately 78,000 female sex workers and their clients (particularly long-distance truck drivers and migrant workers, who are considered “bridge” populations that could enable further transmission of HIV to the wider population), as well as by unprotected sex between about 25,000 men who have sex with men.^7^ In 2007, HIV prevalence among female sex workers in Karnataka (measured primarily at sentinel surveillance sites at antenatal clinics) was 5.3%.^6^ However, in 5 districts of the state surveyed as part of an integrated behavioral and biological assessment, HIV prevalence among female sex workers ranged from 9.5% to 34.2%, and among men who have sex with men in 1 district, prevalence was 19.5%.^8^

Under NACP-III, Karnataka's SACS, called the Karnataka State AIDS Prevention Society (KSAPS), was responsible for HIV prevention, care, and treatment programming across the state, including prevention programming among key populations in 10 of the state's 30 districts. The remaining 20 districts were covered by the Avahan program funded by the Bill & Melinda Gates Foundation.^9^

KSAPS had significant structural limitations, including frequent turnover of senior staff and just 6 departmental heads responsible for all aspects of HIV programming. Moreover, these departmental heads were mainly preoccupied with administrative tasks, leaving them little or no time to provide technical and managerial support to programs or to offer direct support in the field. When NACP-III began in 2007, the state was implementing 18 prevention interventions in its 10 districts. However, it reached only 5% of the approximately 21,000 female sex workers in these districts regularly, and coverage of the around 7,000 men who have sex with men was inadequately documented. Furthermore, routine program monitoring indicators were not being collected or reported from all the interventions. There was a clear need to bolster the capacity of KSAPS to translate national guidance and strategy into action on the ground,^10^ by increasing coverage of key and bridge populations with prevention services, improving the quality of the programs, and ensuring that program indicators were collected and reported for performance monitoring and accountability. In accordance with the national strategy, NACO established a Technical Support Unit in Karnataka in 2008.

## ESTABLISHING THE TECHNICAL SUPPORT UNIT IN KARNATAKA

Following a Request for Applications, NACO awarded the contract to form a TSU to India Health Action Trust (IHAT), an organization affiliated with Karnataka Health Promotion Trust, which was implementing the Gates-funded interventions in 20 districts of Karnataka.

The Bill & Melinda Gates Foundation provided NACO with funding for the first 4 years of the TSU, but the TSU was fully accountable to the state and national government. IHAT signed a Memorandum of Understanding with KSAPS to clarify the TSU's initial roles and responsibilities. KSAPS and NACO reviewed IHAT's contract annually for performance, with the opportunity to reassess goals and objectives. Once the TSU staff had been hired in consultation with the state government, IHAT took primarily a backseat role in order to ensure the TSU's profile as part of the government rather than an as an external organization and in order to make collaboration easier.

The TSU's 18 staff were hired from the private and nongovernmental sectors for their managerial and technical expertise. Senior staff were hired from the Avahan program because of their expertise and experience in Karnataka and to enable a quick transfer of lessons from Avahan, which had achieved greater program coverage in the 20 districts it covered than the KSAPS interventions had in the other 10 districts. Field-level staff were hired from the open market. Positions and salaries were set by the national government. Given the technical and managerial experience of the staff and in order to attract high talent, salaries were slightly higher than government salaries for corresponding positions in KSAPS.

The TSU staff included:

- A team leader to provide overall supervision and guidance
- A strategic planning officer to guide strategy and develop annual plans
- Key population program officers and an advocacy officer to provide supervision and guidance to the implementing NGOs
- Clinical program officers to provide technical support to the clinical components of the programs
- Capacity-building officers to design training
- A program officer for information, education, and communication (IEC)
- An M&E officer to assist field agencies with collecting, uploading, and analyzing monitoring data and to support survey design and implementation

The program officers were placed in districts across the state to make it easier for them to supervise the implementing agencies. The remaining TSU staff worked in the same offices as the KSAPS staff so that the two teams could work in an integrated manner. Reporting relationships between the TSU and KSAPS also facilitated this integration. The TSU's team leader reported primarily to the head of KSAPS and also had a “dotted line” of reporting to NACO's own national-level TSU. The reporting relationship to the head of KSAPS ensured ownership of the TSU's work at the state level, while contact with the national TSU helped ensure that national guidance was translated to the state level. Overall, this reporting arrangement, along with regular reviews of the unit's activities at both the state and national government levels, facilitated coordination and good relations between all the bodies. Below the leadership level, TSU staff reported to the TSU team leader and not to KSAPS staff, but the TSU's technical and program officers worked closely with KSAPS officers (Figure 3).

Most of the Technical Support Unit staff were embedded in the state government office to facilitate close collaboration.

While sharing the same strategic goals, KSAPS was largely responsible for the administrative side (procuring NGOs to run interventions, releasing funding, conducting audits), while the TSU was responsible for the technical side. The two entities thus complemented each other and operated as one to fulfill the HIV prevention mandate of the state.

## TSU SUPPORT

The TSU provided support in 5 key areas, regardless of whether the prevention interventions for key populations were government- or donor-run:

1. Assisting in strategic planning for management, scale up, and monitoring
2. Building and rolling out a comprehensive M&E system for the state
3. Providing direct supportive supervision to intervention units
4. Facilitating training
5. Assisting with IEC

### Strategic Planning

The TSU provided technical input to the state government's Annual Action Plans for HIV prevention, treatment, care, and support.^11^ These plans, submitted by KSAPS to NACO for review and approval, contain the targets, budget, and implementation strategy for the upcoming year. The TSU also helped the KSAPS program officers develop activity plans for each department within KSAPS, to ensure timely implementation and use of funds.

One of the TSU's primary areas of support was to map the districts to identify where there were gaps in coverage and advise KSAPS on how to allocate funds and interventions accordingly. These mapping exercises estimated the denominator of key and bridge populations across the state by typology, defined service areas, recommended locations of sexually transmitted infection (STI) clinics where they would be most accessible to key population members, and set targets to maximize contacts by peer educators. Peer educators are members of key populations recruited to provide outreach to other key population members—namely, to deliver HIV prevention information and commodities and encourage clinic attendance. The peer educators are selected for their knowledge of their communities and their credibility and trust within them, which make them better able to reach their peers than outreach workers who are not members of key populations.^12^

One key activity of the Technical Support Unit was a district mapping exercise to identify gaps in program coverage.

Where mapping showed the need for new interventions, it was the responsibility of KSAPS to procure an NGO. Once the NGO was contracted, the TSU would help the NGO develop its proposal, work plan, and financial plan, as well as support the program for scale up.

### Monitoring and Evaluation

The TSU's role in M&E was to improve data systems so that outputs and program performance could be tracked and accountability systems put in place. The TSU worked with the implementing NGOs to ensure that nationally designed planning and monitoring tools were implemented across the state and that reporting took place on a routine basis. One example is the microplanning tools specially designed to be used by peer educators (including those with no literacy skills) to plan and record their contacts with individual key population members and detail all services provided. Linking outreach with clinical reporting helped the implementing partners understand their performance and use data for decision-making.

The TSU monitored the output of core NACP-III indicators, developed a performance monitoring dashboard for KSAPS leadership, and routinely analyzed and presented data to KSAPS. This allowed for accountability at two levels: (1) the NGOs were accountable to KSAPS, and (2) KSAPS, in turn, was accountable to NACO for overall performance.

The TSU also developed and implemented behavioral surveys (polling booth surveys) to monitor behavioral outcomes of programs, conducted periodic reviews of the NGOs on fiscal and programmatic areas, and updated KSAPS on progress. Official annual evaluations of each intervention were conducted by KSAPS, with input on performance monitoring from the TSU.

### Supportive Supervision

Through its key population officers based in the field and clinical program officers, the TSU provided direct, intensive technical and management support to the interventions. Program officers were decentralized and spent 1–3 days a month with each intervention unit for which they were responsible. They provided support to clinical services, linkages to other services, monitoring systems and reporting (as described earlier), and community mobilization. TSU staff also ensured that KSAPS supplied commodities (including condoms, lubricant, and IEC materials) to the interventions in a timely manner and that implementing NGOs provided adequate documentation and reporting.

### Facilitating Training

The TSU capacity-building officer worked closely with the KSAPS counterpart to identify training institutions and resource people for staff of the intervention units, design training modules and materials, develop an annual training calendar, and evaluate trainings. The TSU trained the staff of STI clinics (referral, static, and outreach) that were contracted by KSAPS, to ensure that they provided appropriate examinations and testing for members of key populations and treated them respectfully. Recruitment and training of peer educators was an additional focus to increase program coverage.

### Information, Education, and Communication

The communications officer supported KSAPS in the design and overall rollout of IEC campaigns for HIV, and especially for key populations. The communications officer also supported the NGOs in rolling out IEC materials at the field level.

## METHODS

**Figure 4.**
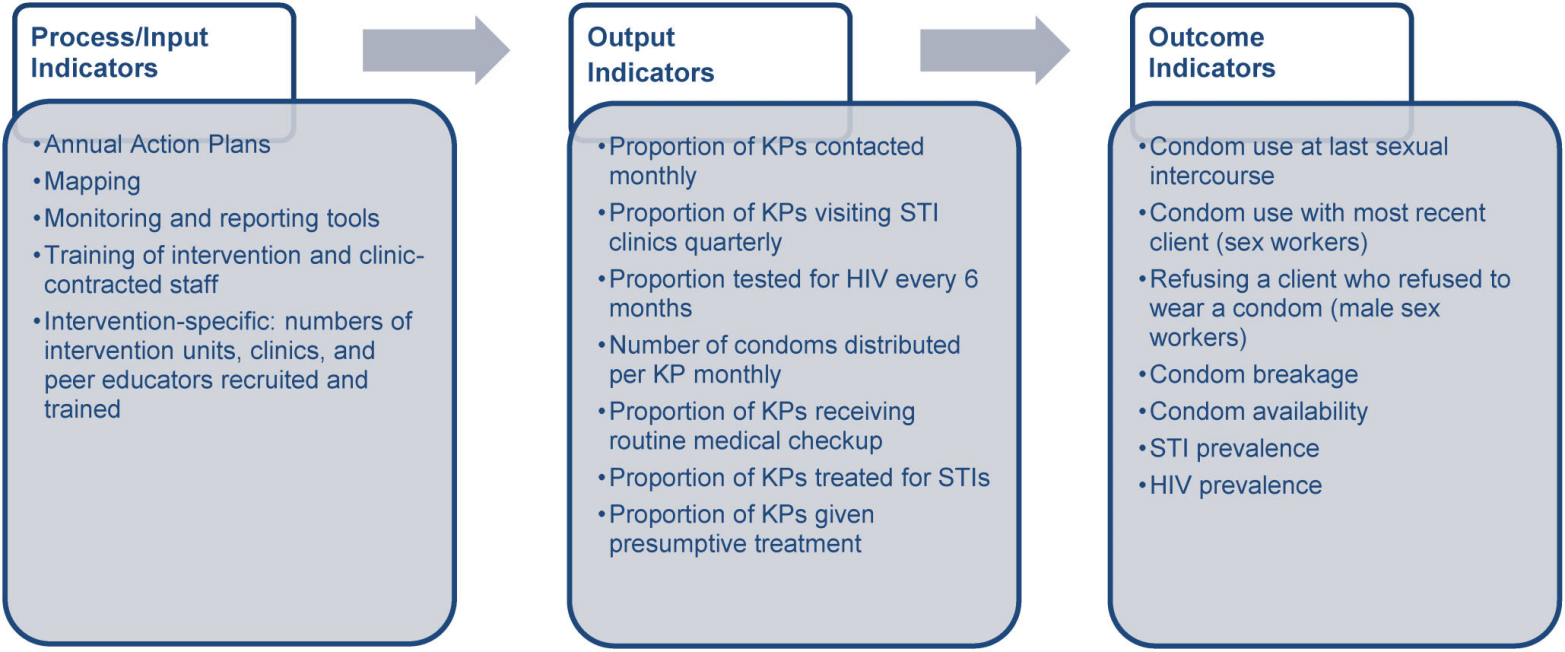
Results Framework for the Karnataka State AIDS Prevention Society and Technical Support Unit, Selected Indicators

We used the results framework for KSAPS and the TSU to assess the effectiveness of the TSU and state government's joint efforts in scaling up HIV prevention programming in Karnataka (Figure 4). Specifically, we focused on 3 types of indicators to measure success: process/input, output, and outcome. KSAPS monitoring data provided information on process indicators, including the number of intervention units, STI clinics, and peer educators. Program monitoring data provided information on key output indicators that give an indication of program coverage; examples of such indicators are the proportion of female sex workers and men who have sex with men who were contacted monthly by the program, the proportion of these key population groups who regularly visited STI clinics and HIV testing and counseling centers, and the number of condoms distributed to these groups. Finally, to monitor changes in key sexual and health-seeking behaviors among key population groups (ie, outcome indicators), the TSU implemented polling booth surveys with female sex workers and informal confidential voting interviews with men who have sex with men. A polling booth survey is a group interview method in which individuals give anonymous responses through a ballot box, while informal confidential voting interviews blend face-to-face interviews with anonymous voting methods.^13^^–^^15^ See the Box for sample indicators measured in the polling booth surveys with female sex workers. These approaches are more suitable than conventional interviewing and surveying methods to collect information on sensitive and personal issues, such as sexual behaviors, particularly among low-literacy populations.

**BOX. Indicators Measured in Polling Booth Surveys With Female Sex Workers**

- Condom use at last sexual intercourse with:
  - Clients (paying new or occasional clients)
  - Regular clients (paying regular clients)
  - Lovers (nonpaying lovers of the female sex workers who do not live with them)
  - Husbands or cohabiting partners (nonpaying husband or live-in partner)
- Sex without a condom
- Condom breakage
- Barriers to using condoms
  - Partner's choice
  - Influence of alcohol
  - Condoms not available
  - Client offers more money for sex without condoms
- Anal sex
- Risk perception
- Experience of violence
- HIV testing
- Knowledge of antiretroviral therapy
- Experience of sexual violence in the past year

## RESULTS

### Process Indicators

The TSU's assistance in **strategic planning with KSAPS through the Annual Action Plans** contributed to the state government's success in increasing the overall KSAPS budget from US$8.0 million in 2007–2008 to $13.1 million in 2011–2012. Over the same period, the portion of the state budget allocated to prevention interventions among key populations tripled, from $0.7 million to $2.1 million, partly because the TSU-facilitated mapping exercises revealed higher numbers of key population members. Planning for the transition of the donor-funded intervention units to KSAPS management also required additional funds.

With assistance from the Technical Support Unit, the Karnataka state government's budget increased from US$8 million to $13 million within 4 years.

The TSU also ensured that national **reporting** formats were implemented. In 2007–2008, only 56% of intervention units in the 10 KSAPS districts had been reporting on a monthly basis, but within 2 years 100% were doing so with TSU-provided training and support, using the national government's comprehensive set of indicators. This, in turn, helped KSAPS understand gaps in programming and develop plans for comprehensive state coverage.

The combined efforts of the TSU and KSAPS led to a **significant improvement in coverage** of key populations in Karnataka through increases in the number of intervention units and STI clinics. Between 2007–2008 and 2008–2009, the number of intervention units in the 10 districts overseen by KSAPS increased from 9 to 26. The number of STI clinics (static, referral, and outreach) in these districts grew from 16 in 2007–2008 to 211 in 2011–2012. In the 4 years from 2009, KSAPS increased its coverage beyond these 10 districts, taking responsibility for the 27 preexisting Gates-run intervention units and adding new units to encompass all districts in the state. As a result, between 2008–2009 and 2012–2013, the number of intervention units managed by KSAPS increased almost fourfold to 126. The ability to absorb donor-funded programs without compromising coverage and quality was key to the long-term sustainability of HIV prevention among key populations throughout the state.^16^

In the initial 10 districts overseen by the Karnataka state government, the number of STI clinics grew from 16 to 211 between 2007 and 2012.

Meanwhile, the number of peer educators for female sex workers statewide increased from 345 in 2008–2009 to 621 in 2011–2012, and for men who have sex with men, from 86 to 326.

### Output Indicators: Program Coverage

**Figure 5.**
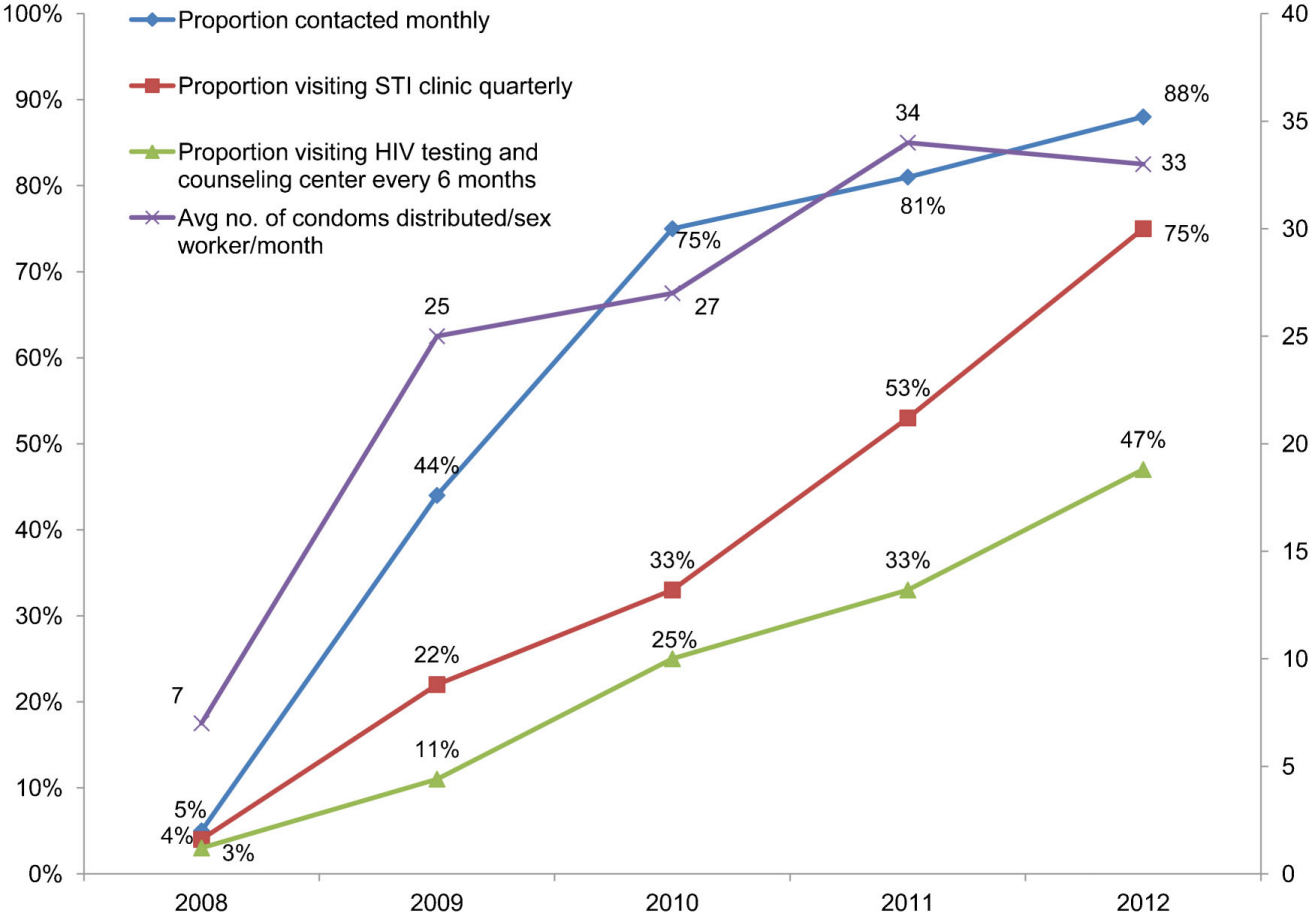
Coverage of Mapped Female Sex Workers (N = 20,806 ) by State Government HIV Prevention Programs, 10 Districts of Karnataka, 2008–2012 Targets: 80% of mapped number contacted monthly; 100% visiting STI clinic quarterly; 100% receiving HIV test every 6 months; average of 32 condoms per female sex worker distributed monthly. Source: Karnataka State AIDS Prevention Society monitoring data.

**Figure 6.**
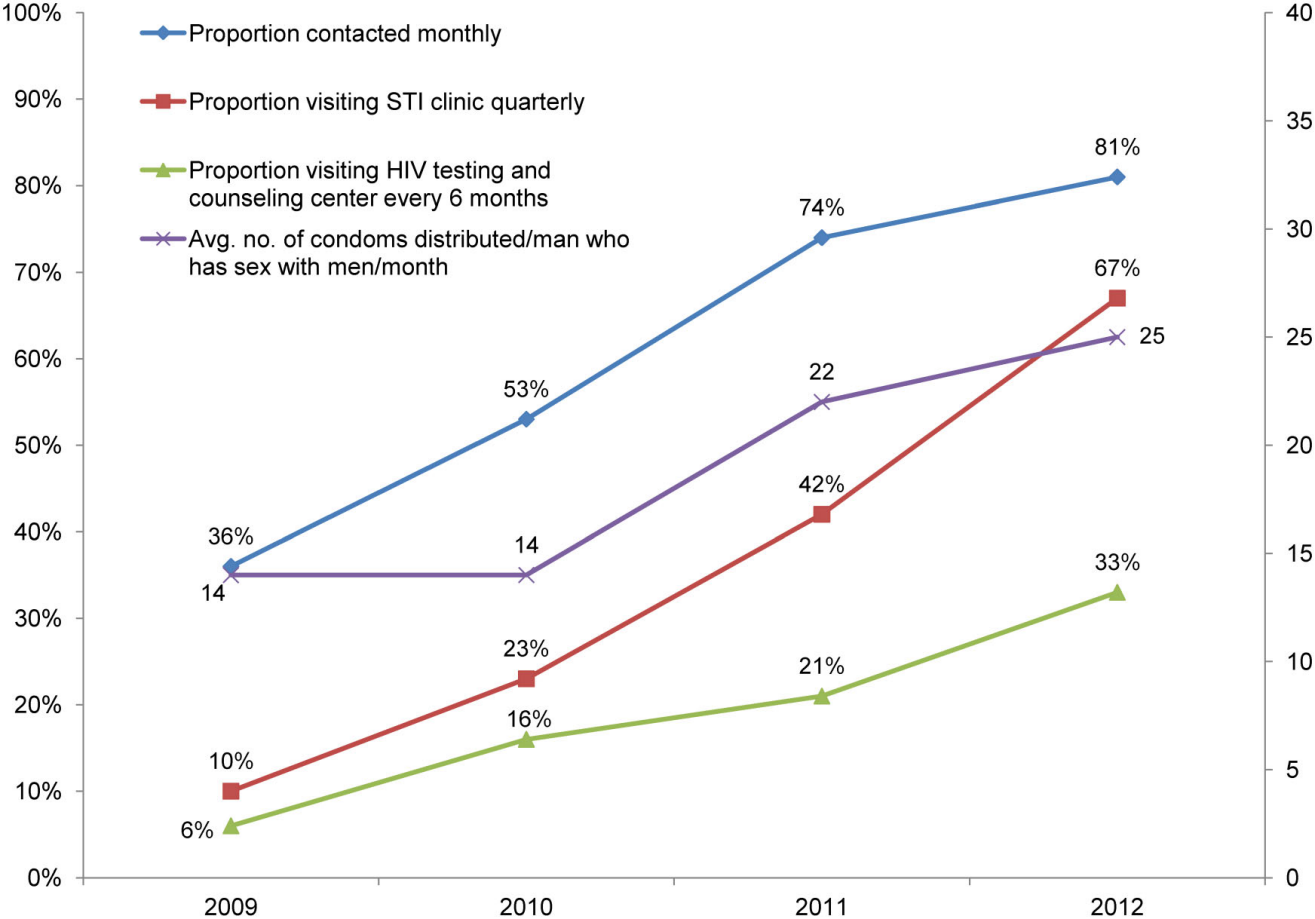
Coverage of Mapped Men Who Have Sex With Men (N = 7,054) by State Government HIV Prevention Programs, 10 Districts of Karnataka, 2009–2012 Targets: 80% of mapped number contacted monthly; 100% visiting STI clinic quarterly; 100% receiving HIV test every 6 months; average of 24 condoms per man who has sex with men distributed monthly. Source: Karnataka State AIDS Prevention Society monitoring data.

The increased number of peer educators was associated with enhanced program coverage and an improvement in output indicators for both female sex workers and men who have sex with men. For example, the proportion of female sex workers contacted monthly by the program increased from an average of 5% in 2008 to 88% in 2012 (Figure 5); among men who have sex with men, the increase was from an average of 36% in 2009 to 81% in 2012 (Figure 6). (The target set by NACO for both populations was 80%.) By 2012, 75% of female sex workers and 67% of men who have sex with men were visiting an STI clinic quarterly. Although these levels were below the NACO target of 80%, they increased considerably from the 2008 levels of 4% and 7%, respectively. The proportion of female sex workers visiting an HIV testing and counseling center every 6 months increased from 3% to 47% between 2008 and 2012, while for men who have sex with men, the corresponding increase was from 6% to 33%. The average number of condoms distributed monthly per female sex worker in 2012 was 33, above the NACO target of 32, and the corresponding figure for men who have sex with men was 25, exceeding the target of 24. (Lubricants were also distributed along with condoms, although NACO did not require recording this.)

Monthly contact with female sex workers by the state program increased from 5% to 88% between 2008 and 2012.

### Outcome Indicators: Sexual Behaviors

**Figure 7.**
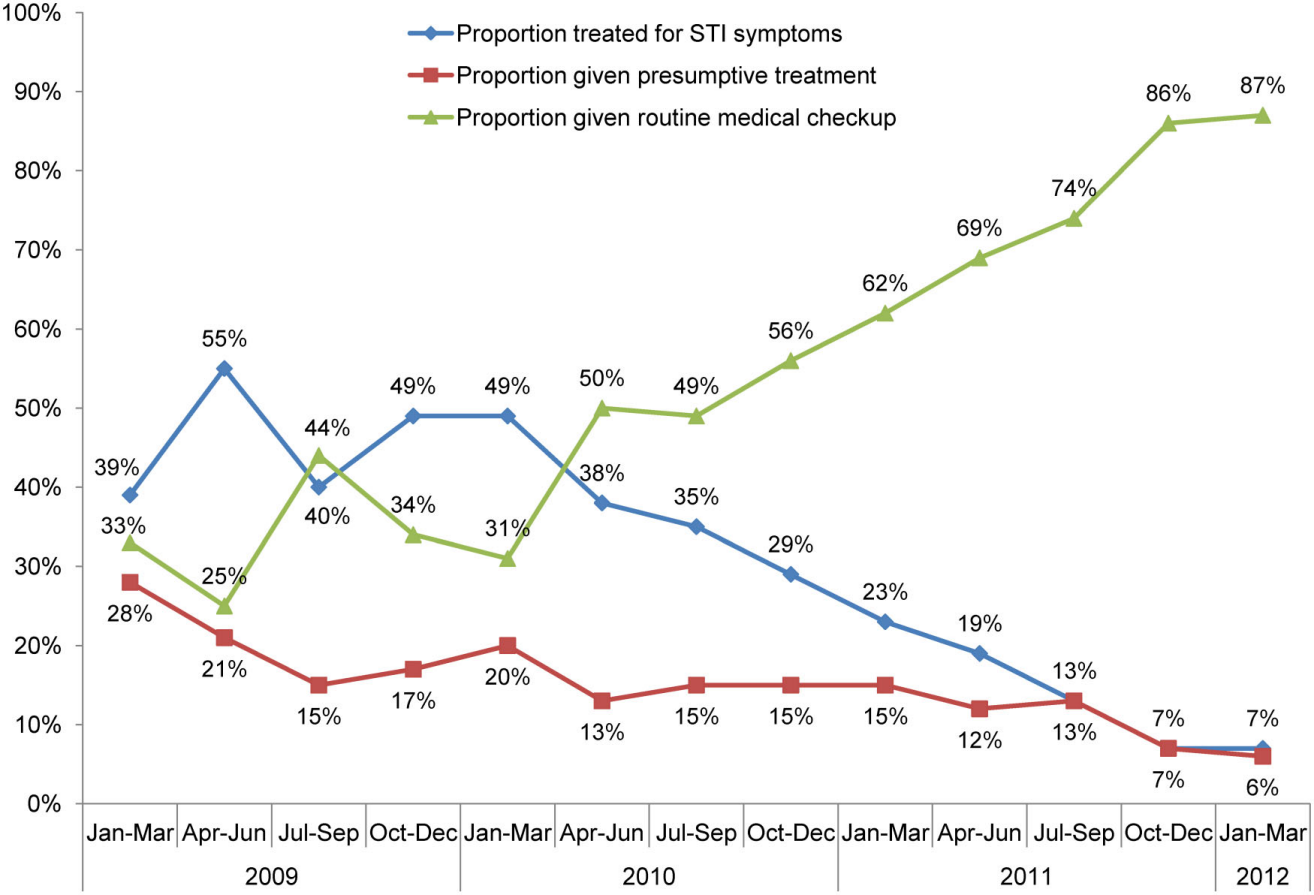
Medical Treatment of Female Sex Workers by State Government HIV Prevention Programs, 10 Districts of Karnataka, 2009–2012 Source: India Health Action Trust monitoring data.

In 9 of the 10 KSAPS program districts, the overall proportion of female sex workers reporting that they used a condom at last intercourse rose from 60% in 2008 to 68% in 2010; over the same period, the proportion reporting that their most recent client used a condom grew from 71% to 78%. The percentage of female sex workers reporting that a condom broke the last time they tried to use one declined from 26% to 14%, and those prevented from using a condom at last sex because it was not available fell from 27% to 20%. Program data showed that while the proportion of female sex workers receiving routine medical checkups increased substantially from 33% in the first quarter of 2009 to 87% in the first quarter of 2012, the proportion treated for STI symptoms declined from 39% to 7% over the same period (Figure 7).

Sexual behaviors of key populations, such as using a condom at last sex, improved over time.

**Figure 8.**
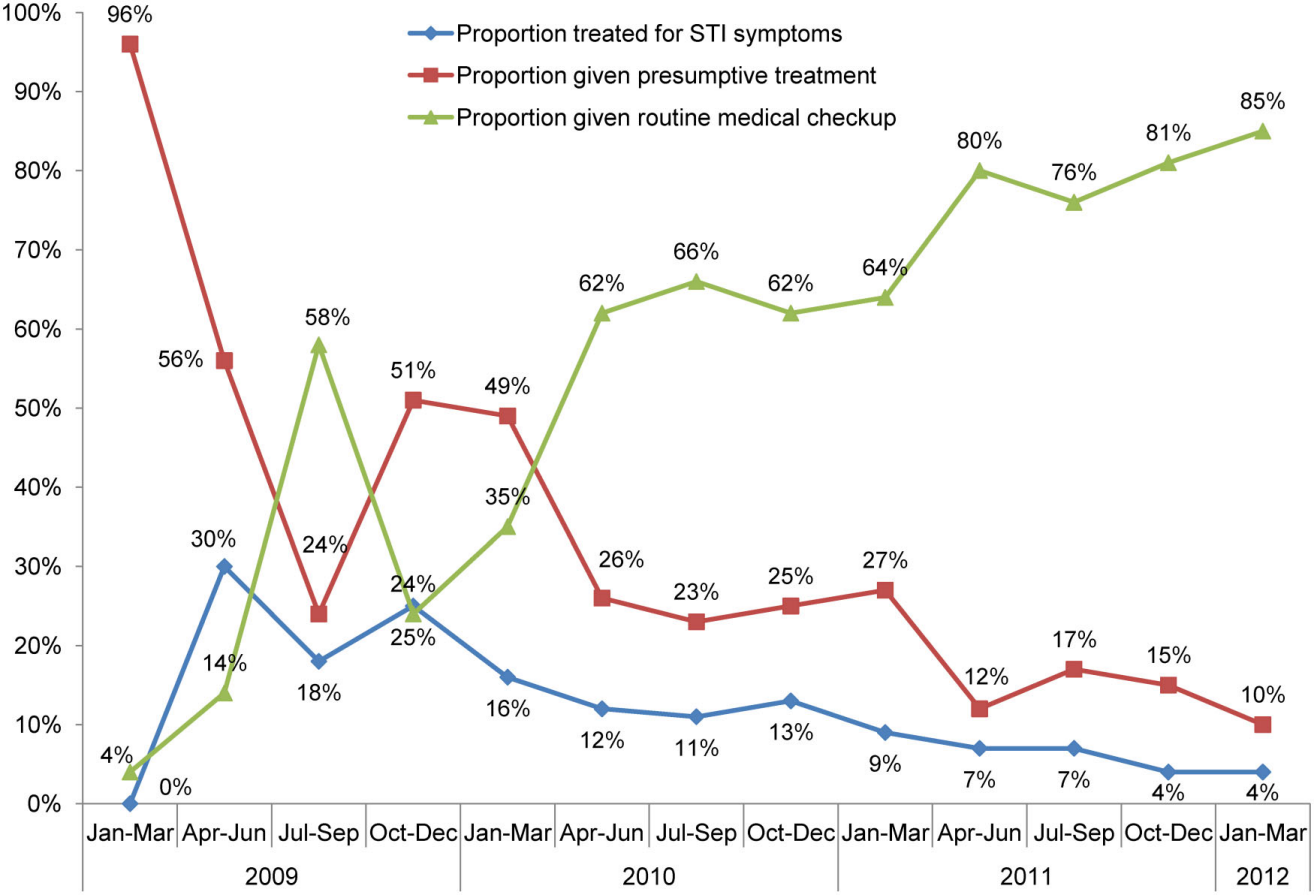
Medical Treatment of Men Who Have Sex With Men by State Government HIV Prevention Programs, 10 Districts of Karnataka, 2009–2012 Source: India Health Action Trust monitoring data.

Between 2008 and 2010, the proportion of men who have sex with men in 6 program districts who reported using a condom at last anal sex increased from 89% to 97%, and the percentage using a condom at last anal sex with their regular male partner rose from 76% to 86%. The proportion of male sex workers refusing to have anal sex because a client refused to use a condom rose from 45% to 72%. As with female sex workers, the proportion of men who have sex with men being treated for STI symptoms declined, from 30% in the second quarter of 2009 to 4% by the beginning of 2012, even as the proportion receiving routine medical checkups grew from 14% to 85% over the same period (Figure 8).

## DISCUSSION

Because of the complexity of the programming environment in Karnataka, with multiple actors including KSAPS, the TSU, and the Bill & Melinda Gates Foundation, among others, it is not possible to attribute statewide HIV prevention outcomes directly to the TSU's work. However, in the 10 districts overseen by KSAPS, there were changes after the TSU was established, and it seems reasonable to infer that the technical assistance given by the TSU may have contributed to KSAPS' success in improving outcomes.

The Technical Support Unit likely contributed to the improved outcomes of the state government HIV prevention program.

The TSUs are part and parcel of the national strategy to rapidly scale up HIV prevention programs among key populations, and they are integral to implementation of the strategy. In Karnataka, the experience has been that a TSU of experts with clear deliverables and systems for close coordination with the government has ensured a common sense of mission and cohesion.

We would like to emphasize that the increased outputs described earlier likely occurred as a result of the collaboration between the TSU and KSAPS. The results should therefore not be seen as due to the success of the TSU alone, but rather as progress for the state as a whole, using an innovative management model designed to enhance the effectiveness of components of the national government's plan. These components included increased funding, a strengthened reliance on M&E systems, and scale up of interventions to improve coverage.

A number of lessons can be drawn from the Karnataka experience that may be applicable in other contexts where TSUs are being considered.

### Clearly Define the Mandate, Roles, and Responsibilities for the TSU, As Well As Indicators of Success

The formation of TSUs in each Indian state was mandated by the national government, but a Memorandum of Understanding between IHAT (the agency contracted to establish the TSU) and the state government was essential to ensure that both sides understood what the TSU was to do—and, just as importantly, what it was not to do. A Memorandum of Understanding gives the TSU legitimacy, protects it from political or bureaucratic changes within the state government, and allows it to stay focused on its work and deflect additional demands.

In order to be viewed as an unbiased party, the TSU cannot be involved in any decisions about selecting NGOs to implement programs. Its focus should remain on providing technical support once the implementing NGOs have been chosen. Nevertheless, because the TSU's role often involves asking challenging questions and taking difficult decisions about the NGOs, it can be susceptible to political pressure. The Memorandum of Understanding, together with regular reviews at the state and national level, ensures that such matters can be arbitrated when necessary.

### Choose the Right Organization to Manage the TSU

The organization contracted by the national government to hire, train, and compensate the TSU staff must have the appropriate skills and experience in the area of HIV prevention management. It should also have the political maturity required to navigate the complexities of placing staff within the government system and to work with state government officials to create an enabling environment for the TSU to work effectively.

### Do Not Lose Sight of the TSU's Role in Supporting the Government

A TSU can only succeed if its relationship with the government agency is mutually supportive and noncompetitive. The unit must be sanctioned at the highest political levels, and it should be seen as a catalyst to help achieve shared program goals. The TSU's leader should report to the highest state government official to ensure it gets appropriate visibility and support. However, the TSU should not forget that its role is to provide support to the government and that ultimate decision-making power must rest with the government.

Cultivating a mutually supportive and noncompetitive relationship between the Technical Support Unit and the state government agency is crucial.

Clearly defined roles and relationships enhance the TSU's productivity and make it more effective than the ad hoc placement of experts in individual government departments. By working to create an environment of trust, the TSU helps to ensure that its staff will be consulted by state agency staff before they make decisions. Having the TSU staff in the same building as the agency builds this trust and helps the state agency staff feel that they have joint ownership of the TSU's work.

A related point is the need to be sensitive to concerns about salary differentials between the TSU and the government unit. Attracting high levels of technical ability from the private and nongovernmental sectors at competitive rates implies that TSU staff often earn more than their government counterparts, which may cause resentment and can interfere in having a productive partnership. These sensitivities may be mitigated if the TSU does not try to garner praise for itself and shares the credit for any accomplishments with the government.

### Be Sensitive to Costs and Value Added

Particularly in resource-constrained settings, a TSU may be an effective way to leverage government funding to ensure scale and quality in programming. In 2012–2013, the KSAPS prevention budget was US$3.7 million (out of a total budget of $14.3 million), of which $0.7 million (19%) was allocated to the TSU. This helped ensure that the remainder of the prevention budget was spent as effectively as possible.

Governments are often under pressure to produce “quick wins,” so the TSU should aim to demonstrate quickly that it adds value to the government's work. One such area in Karnataka was monitoring, an area that is often weak in programs and one in which it therefore may be possible to produce rapid improvements. Setting up a data collection, analysis, and reporting system to measure outcomes helped the government value the TSU for the knowledge and skills that it brought and for the value that it added to the overall HIV prevention program. One example is the use of polling booth surveys to monitor outcomes, and we would further recommend the adoption of such surveys by TSUs in other states as well.

There were other, less tangible but valuable benefits to the TSU. Although it was not in the remit of the TSU staff to train their government counterparts, they found that by being embedded in the government offices and working alongside them, the TSU staff indirectly helped build the capacity of their government colleagues. Occasionally going beyond the strict terms of the mandate to help government colleagues can build a great deal of goodwill. In Karnataka, although the TSU was focused on HIV prevention programs among key populations, it helped the government write guidelines on STIs (for both key and general populations) and helped develop an HIV capacity-building plan as well as broad IEC activities.

In addition, given the high turnover and staff vacancies that frequently affects government programs, a TSU can offer the additional value of institutional memory, helping to provide much-needed continuity and stability for implementing agencies as well as helping to foster their success.

### Understand Insourcing of Skills as Integral to Building Sustainable Government Health Systems

Some critics assert that “sourcing-in” the key functions of the government to staff who are not core government employees delays much-needed efforts to build health systems and is not sustainable in the long run. We would argue that TSUs should rather be seen as part of a broad approach to strengthening systems and to expanding existing government capacity to manage and scale up health programs. Any such approach should take into consideration the degree of urgency for program implementation, the feasibility of the models for the given context and problem, and the available timeframe for addressing issues of sustainability. In India, the national program discerned an urgent need to improve the range and depth of technical expertise in the states, and it decided that working to strengthen capacity among existing SACS staff would take too long. In low-, middle-, and high-income countries, the private sector often subcontracts other agencies to perform key skills that they might otherwise not be equipped to do. We see no reason why government systems should not be equally creative in their approach, given the complex range of skills needed for health programs.

Technical Support Units should be seen as part of a broad approach to strengthening systems.

We agree that sustainability is a major issue that needs to be addressed, and we propose two ways to look at this issue. The first question should be: Will the TSU be required on an indefinite basis, or is the need for it time-limited? In the case of India, TSUs were acknowledged as critical structures that enabled the scale and quality of HIV prevention programs that was needed for the duration of NACP-III (2009–2012). The second question is: If the TSU is needed for the long term, will the functions it carries out be required at the same level of intensity, or is it possible to move to a less intense model? TSUs have been retained as part of NACP-IV, although in some states, including Karnataka, only a reduced core of staff was maintained, since scale had already been achieved and the emphasis is now mostly on maintaining quality. So, for example, the ratio of field staff to the number of prevention units was reduced from 1:10 to 1:15. Although most of the TSUs in India are now funded by the national government through the World Bank, the Karnataka TSU continues to be funded by the Bill & Melinda Gates Foundation.

It is worth noting that the TSU model not only continues as a national policy within HIV programming in India but also has been adapted for other health programs both within India (such as maternal and child health in the states of Bihar and Uttar Pradesh and for the national immunization program) and externally, such as the Kenyan government's HIV program.

The Technical Support Unit model has been adapted for other health programs within India, as well as in other countries.

### Limitations

We recognize that it is not possible to conclusively attribute the improvements in prevention coverage and service provision among key populations to the formation of the TSU (and as we have indicated earlier, we would not seek to do so, since the TSU and KSAPS worked closely together). A comprehensive evaluation of the TSU is beyond the scope of this article, and a randomized controlled trial of technical assistance was never designed in India and is not feasible. Such a trial would have required comparing Karnataka with a state of a similar size and demographic profile that had no TSU, but all such states in India were mandated by the national government to adopt TSUs at the same time as Karnataka.

## CONCLUSION

The scale up of national health programs requires a broad continuum of capabilities, including policy guidance, budgetary planning and disbursement, technical and managerial support for the implementation of programs, and monitoring and evaluation. In many low- and middle-income countries, it is a challenge to cover this range of skills adequately within a single government agency, but without such capacity it is difficult to scale up programs while maintaining quality. The experience in Karnataka state in India suggests that TSUs—a deliberate partnership with expertise from the nonprofit and private sectors—can be an effective way of enabling rapid scale up while maintaining program quality. A comprehensive and comparative analysis of all TSUs in India is warranted and would be highly valuable to the development field.

## Notes

**Peer Reviewed**
